# Reduced Adenosine Uptake and Its Contribution to Signaling that Mediates Profibrotic Activation in Renal Tubular Epithelial Cells: Implication in Diabetic Nephropathy

**DOI:** 10.1371/journal.pone.0147430

**Published:** 2016-01-25

**Authors:** Catalina Kretschmar, Carlos Oyarzún, Cristopher Villablanca, Catherinne Jaramillo, Sebastián Alarcón, Gustavo Perez, Montserrat M. Díaz-Encarnación, Marçal Pastor-Anglada, Wallys Garrido, Claudia Quezada, Rody San Martín

**Affiliations:** 1 Institute of Biochemistry and Microbiology, Science Faculty, Universidad Austral de Chile, Valdivia, Chile; 2 Nephrology Service Fundació Puigvert, Biomedical Research Institute Sant Pau (IIB Sant Pau), Barcelona, Spain; 3 Institute of Biomedicine and Oncology Programme, National Biomedical Research Institute of Liver and Gastrointestinal Diseases (CIBER EHD), Department of Biochemistry and Molecular Biology, University of Barcelona, Barcelona, Spain; INSERM, FRANCE

## Abstract

Altered nucleoside levels may be linked to pathogenic signaling through adenosine receptors. We hypothesized that adenosine dysregulation contributes to fibrosis in diabetic kidney disease. Our findings indicate that high glucose levels and experimental diabetes decreased uptake activity through the equilibrative nucleoside transporter 1 (ENT1) in proximal tubule cells. In addition, a correlation between increased plasma content of adenosine and a marker of renal fibrosis in diabetic rats was evidenced. At the cellular level, exposure of HK2 cells to high glucose, TGF-β and the general adenosine receptor agonist NECA, induced the expression of profibrotic cell activation markers α-SMA and fibronectin. These effects can be avoided by using a selective antagonist of the adenosine A_3_ receptor subtype in vitro. Furthermore, induction of fibrosis marker α-SMA was prevented by the A_3_ receptor antagonist in diabetic rat kidneys. In conclusion, we evidenced the contribution of purinergic signaling to renal fibrosis in experimental diabetic nephropathy.

## Introduction

Diabetes mellitus is produced by a chronic metabolic unbalance and its incidence rate has dramatically increased worldwide [1]. Diabetic nephropathy (DN) is a long-term complication affecting up to 30–40% of diabetic patients and is the major cause of end-stage renal disease worldwide [2]. DN is also a major risk factor for cardiovascular disease and can be a life threatening condition. The economic and social impact of the illness is high, due to the fact that DN predisposes patients to undergo organ replacement therapies at advanced stages [3,4]. To date, strict controls of glycaemia and arterial blood pressure as well as the use of blockers of the renin-angiotensin system have only been modestly effective in slowing the evolution of DN [5]. Thus, DN remains incurable and there is an urgent demand for new therapies.

Incipient DN is characterized by the occurrence of podocytopathy and alterations in the filtration barrier which are clinically evidenced by hyperfiltration and microalbuminuria. As the disease advances an extensive renal fibrosis arises, characterized as a progressive and irreversible process [6]. A key event in this process is the transition of epithelial cells to a mesenchymal phenotype (EMT) acquiring the properties to mediate renal fibrosis and probably migrate to the interstitium. Typically, the expression of alpha smooth muscle actin (α-SMA) and fibronectin have been used as markers of profibrotic cells activation. Emerging evidence has shown that cytokines such as transforming growth factor-beta 1 (TGF-β), high glucose levels and advanced glycation end products influence profibrotic activation in kidney cells phenotype [7,8]. The discovery of mediators of this pathogenic process is a major current focus due to their valuable therapeutic potential.

Recently, it was demonstrated that a systemic rise of plasma adenosine and derived metabolites occurring in DN patients correlate with the progression of the disease, while the nucleoside levels were basal in diabetic patients without renal complication similar to those observed in healthy people [9,10]. Extracellular adenosine arises from the adenine nucleotide metabolism, thus becoming a crucial step the hydrolysis of AMP [11]. In addition, the equilibrative nucleoside transporters (ENTs), recognized by their sodium-independent facilitative transport activity, mediates the adenosine uptake to be metabolized intracellularly whereas nucleoside can be accumulated outside when the transport activity is reduced. Consequently, it has been described that ENTs could play a major contribution to adenosine availability for autocrine or paracrine signaling through adenosine receptors [12–22]. Notably, the transition from the epithelial to the mesenchymal phenotype was shown in the renal epithelial tubular cells line HK2 knocked down for the equilibrative nucleoside transporter member 1 (ENT1) [23]. These observations suggest a link between altered adenosine handling and profibrotic activation of cells. Adenosine modifies cell function by signaling via the P_1_ purinoceptor family [24]. While A_1_ and A_2A_ subtypes have high affinity for their ligand, the A_2B_ and A_3_ receptors require increased levels of adenosine to mediate a cellular response. Functionally, a protective role for adenosine A_2A_ receptor subtype in diabetic nephropathy by attenuating inflammatory responses has been described [25]. Absence of A_1_ adenosine receptor results in an increase of both the diabetic hyperfiltration and the glomerular damage, thus suggesting that it also plays a protective task [26,27]. In contrast, the A_2B_ receptor subtype mediates pathogenic events that trigger diabetic glomerulopathy [22]. While a role for the A_3_ receptor subtype has been recently associated with the progression of renal fibrosis in a model of chronic kidney disease [28], its contribution to the pathogenesis of diabetic renal disease remains to be analyzed.

The goal of this study was determine the mechanism that mediates altered adenosine handling in the diabetic kidney and its relation with purinergic signaling and renal fibrosis. We provide evidence of a decreased adenosine uptake occurring in the epithelial tubule cells of the diabetic kidney concomitant with the progression of renal disease. We also demonstrate the contribution of adenosine receptor signaling to the phenotypic transition of cells.

## Material and Methods

### Materials

Pharmacological modulators of adenosine receptors and inhibitors of nucleoside transporters were purchased from Tocris Biosciences. Culture mediums, serum, antibiotics and supplements were purchased from Invitrogen. Chemicals and streptozotocin were purchased from Merck KGaA. Tritiated adenosine was obtained from American Radiolabeled Chemicals.

### Cell culture

Human proximal epithelial tubule cell line (HK2) was obtained from ATCC. The cells were propagated in Keratinocyte Serum Free Medium (K-SFM) supplemented with 0.05 mg/ml of bovine pituitary extract (BPE) and 5 ng/ml human recombinant epidermal growth factor (EGF) and antibiotics, at standard condition of 5% CO_2_ at 37°C in a humidified atmosphere.

### Animal models

Diabetes was induced in male rats (Sprague-Dawley) weighing 250 g by single intravenous administration of streptozotocin (STZ) at 55 mg/kg dissolved in citrate buffer, pH 4.5. Controls rats were injected with an equivalent volume of vehicle. The diabetic groups included animals presenting blood glucose levels ≥ 25 mmol/L. Animals were monitored up to four months. Also diabetic rats were treated with the adenosine A_3_ receptor antagonist MRS1220 at doses 0.1 mg/kg via intraperitoneal administration from days 31 to 60 post diabetes induction, and animals were then sacrificed. *Ent1* knockout mice were originally generated by Choi et al. [16]. Renal samples were obtained from the University of Barcelona´s own colony.

### Ethics statement

All animal procedures were approved by the Institutional Committee on the Use of Live Animals in Research at the University Austral de Chile (Ref. 2012/59). Animal welfare was monitored daily and all efforts were made to minimize suffering. Any animal showing signs of limited movement or significant weight loss were euthanized by intra-peritoneal injection of sodium thiopental (200 mg/kg body weight).

### Human biopsies

Human renal samples were obtained from patients controlled in the nephrology department of Fundació Puigvert, Barcelona, Spain. Use of samples was approved by the Comité Ético de Investigación Clínica (CEIC) from Fundació Puigvert (Ref. 2010/19). Percutaneous kidney biopsies from 9 diabetic patients with nephropathy and normal kidney tissue from 3 patients affected by renal carcinoma were used for the study. The biopsies were obtained for diagnosis in all patients before 2005, without further follow up in Fundació Puigvert, the study was submitted to ethic committee considering that following the current legislation, were not required informed consent for participate in this study.

### Proximal convoluted tubule (PCT) isolation

Renal PCT were extracted from healthy and diabetic rats as described previously [29]. The kidneys were perfused through the renal artery, first with Hank's Balanced Salt Solution (HBSS) and then with HBSS containing 1mg/ml collagenase type II. Renal cortical slices were incubated for 10 minutes with HBSS/collagenase and maintained at 37°C, 50 mg/ml BSA was then added, maintaining the agitation for another 5 minutes. The homogenate was poured through gauze to eliminate non digested tissue. The extracts were centrifuged at 300 x *g* for 3 minutes and then the pellets were washed with HBSS without CaCl_2_ and centrifuged. The remaining material was resuspended in 45% percoll (GE Health Care) in solution HBSS without CaCl_2_ and centrifuged at 7900 x *g* for 15 minutes at 4°C, obtaining 3 phases. The middle phase, containing the PCT was extracted and washed. The pellet was diluted between 8 to 14 times depending of the amount. The PCT extracts were used immediately for transport activity assays. Purity of extracts was assessed by detecting the enrichment of the PCT marker aquaporin 1 and loss of glomerular nephrin and podocin.

### Nucleoside transport activity

HK2 cells were cultured in 24 well plates in presence of 5mM or 25mM glucose for 24 h. Nucleoside transport activity was assayed in choline buffer (in mM: 5.4 KCl, 1.8 CaCl_2_, 1.2 MgSO_4_, 10 Hepes, 137 choline chloride, pH 7.4) supplemented with [^3^H]-adenosine (2mCi/nmol) and 10μM of adenosine at 22°C. Incubation was stopped after 20 seconds by washing the monolayers twice in 2 ml of cold buffer composed of 137 mM choline chloride and 10 mM Tris-Hepes, pH 7.4. Cells were then lysed in 0.250 ml of 1M NaOH. Aliquots were taken for protein determination and for radioactivity counting. Particular uptake rates for equilibrative nucleoside transporters 1 or 2 (ENT1, ENT2) were assigned to transport activities which were inhibited by 1 μM S-(4-nitrobenzyl)-6-thio-inosine (NBTI) or 2 mM hypoxanthine, respectively [30]. Total nucleoside uptakes in cells mediated by concentrative and equilibrative systems were also measured using transport buffer containing sodium chloride. Sodium-dependent uptake rates were obtained by subtracting adenosine uptakes in choline buffer to the total adenosine uptakes in buffer containing sodium chloride.

### Nucleoside transport activity in PCT

Freshly purified PCTs were incubated in 100μl of choline solution with or without NBTI or hypoxanthine for 30 minutes. Nucleoside transport activity was assayed in choline buffer supplemented with or without NBTI or hypoxanthine, with [^3^H]-adenosine (2mCi/nmol), and 20 μM of adenosine. The uptake of adenosine was assayed adding 100μl of solution with [^3^H]-adenosine for 20 seconds at 22°C. Incubation was stopped by washing with 1ml of cold buffer composed of 137 mM choline chloride and 10 mM Tris-Hepes (pH 7.4). Cells were then centrifuged at 12000 x rpm for 5 minutes at 4°C and washed again. After the second wash the pellet was dissolved in 250μl of 0.5M HCOOH. Aliquots were sampled for protein determination and for radioactivity counting.

### Adenosine quantification

HK2 cells were cultured at 80% confluence under standard conditions in 6 well plates for 24 h. The medium was then replaced for Tyrode buffer (10mM HEPES, 12mM NaHCO_3_, 137mM NaCl, 2.7mM KCl, 5mM Glucose and 1mM CaCl_2_) supplemented with 2.5μM EHNA and 1μM NBTI for 1h. The adenosine content was quantified using derivatization with 2-chloroacetaldehyde and HPLC with fluorometric detection [20]. The values were normalized to the total amount of cellular proteins. For adenosine quantification in plasma of rats, samples of blood were extracted in proportion 3:1 with STOP solution (10mM EDTA, 10mM EGTA, 1 mM dipyridamole and 2.5 μM EHNA) from caudal vein. Plasma samples were deproteinized using 10% TCA, neutralized with 2 N KOH and treated with solution ZN/Ba [10]. Finally, samples were derivatized and quantified as described above.

### Protein extracts

Total proteins were extracted with lysis buffer (2% SDS, 10% glycerol, 63.5 mM Tris HCl, pH 6.8) containing Complete Proteinase Inhibitor and 1μg/ml pepstatin (Roche). The proteins were quantified using BCA protein assay kit (Thermo Scientific).

### Western blots

Protein extracts (50μg) were fractionated by 10% SDS-PAGE and transferred to PVDF membranes. The blots were washed with wash buffer (PBS1x, 0.05% Tween20), blocked for 1 h with 0.1% BSA, and incubated with primary antibodies anti-fibronectin (SC-56391), anti-α-SMA (SC-130617) or anti-Pro-COL1A2 (SC-8787) from SantaCruz Biotechnology, and anti-ENT1 from Protein Tech (II 337-1-A7). Primary antibodies against adenosine receptors were anti-A_1_AR from Sigma, anti-A_2A_AR and anti-A_2B_AR from Abcam and anti-A_3_AR from Millipore. The membranes were washed and the primary antibodies were detected using HRP-coupled secondary antibodies. A chemiluminescence procedure was used for the detection of the proteins (Thermo Scientific). The protein levels were expressed as the ratio between the target protein and β-actin or tubulin detected in the same membrane.

### siRNA

Adenosine A_3_ receptor subtype knockdown was achieved in HK2 cells using commercially available and validated short interfering RNAs (siRNA) from Ambion catalogue number AM16708. Cells seeded in 6-well plates (200,000) were transfected with 100 pmol of siRNA using lipofectamine 3000. Following 24 h post-transfection the cells were exposed to transdifferentiation stimuli and the expression of fibronectin was evaluated by western blot.

### Immunohistochemistry

Rat and human kidney tissues were fixed in formalin, embedded in paraffinand 5 μm sections were mounted on silanized slides. Immunodetections were performed as described previously [20] using the primary polyclonal anti-ENT1 antibody from Protein Tech (II 337-1-A7) and the monoclonal anti-αSMA (sc130617) and anti-A_3_AR (sc-13938) from Santa Cruz Biotechnology. The immunosignals were detected using the LSAB+ System–HRP (DakoCytomation).

### Statistical methods

Values are means ± SD, where n indicates number of animals used or number of experiments in cells each one in triplicates. Statistical analyses were carried out on raw data using the Peritz F multiple means comparison test. Student’s t-test was applied for unpaired data.

## Results

### Diabetes modifies extracellular adenosine metabolism in epithelial tubule cells

**Fig 1:**
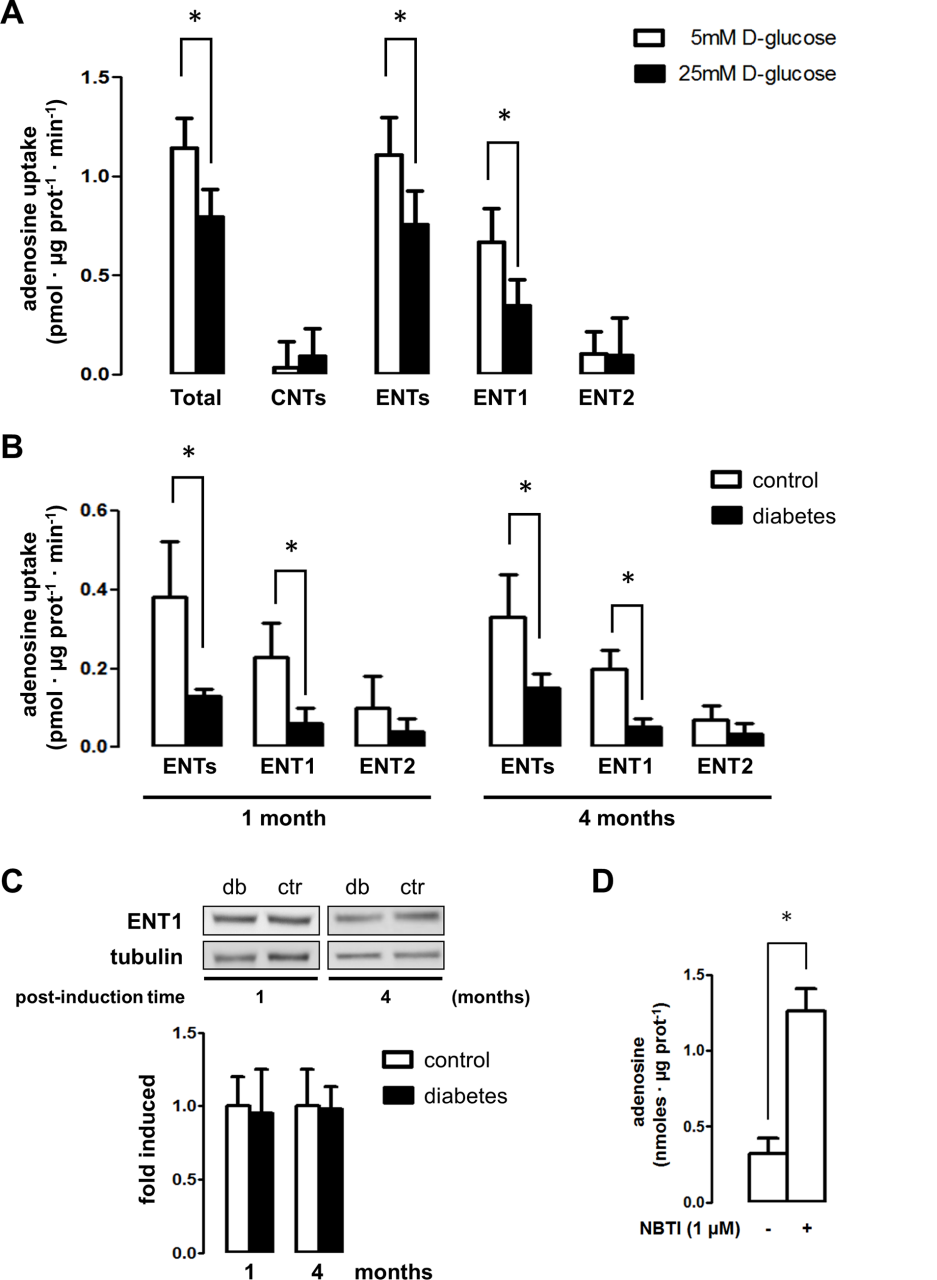
Nucleoside transport activity in renal epithelial tubule cells. A. Total and particular [^3^H]adenosine transport activities were assayed in HK2 cells. The effect of D-glucose concentration on transport activities was assayed in HK2 cells exposed to 5mM and 25mM for 24h. Total uptake activity mediated by concentrative (CNTs) and equilibrative (ENTs) systems was obtained in transport buffer containing Na^+^; while ENTs-mediated uptake was determined by using a Na^+^ free buffer. CNTs component was derived from the difference between total transport activity in Na^+^ containing buffer minus the transport activity in Na^+^ free buffer. The graphs depict particular ENT1 mediated nucleoside uptake as the fraction of the transport in Na^+^ free buffer inhibited by 1μM NBTI, while the ENT2 fraction that was inhibited by 2mM hypoxanthine. Data is expressed as mean ± SD of triplicate measurements from 20 independent assays. * *P* < 0.05 versus 5mM D-glucose. B. Renal proximal tubules were isolated from vehicle- and STZ-treated rats following 1 and 4 months. The effect of diabetes on sodium-independent transport uptake activities mediated by ENT1 and ENT2, was quantified. Data is expressed as mean ± SD of triplicate measurements for assays using extracts from 5 animals in each group. * *P* < 0.05 versus control. C. The protein level of ENT1 in tubule extracts was evaluated by western blot. Representative images of western blots using diabetic (db) and control (ctr) rat extracts are shown. The graph depicts the mean ± SD of ratio between immune signals of ENT1 vs tubulin. The ratio in control extracts were normalized to 1. A statistically significant difference was not found. n = 6. D. The extracellular adenosine levels were quantified in the medium of HK2 cells exposed to NBTI, an inhibitor of ENT1 activity. Data is mean ± SD from 5 experiments. * *P* < 0.05 versus control.

In order to characterize the mechanisms that control extracellular levels of adenosine, we analyzed the nucleoside uptake rates mediated by the nucleoside transporters in epithelial tubule cells. Using the epithelial cell line HK2, derived from human proximal tubules, it was determined that the adenosine uptake activity was largely mediated by the equilibrative sodium-independent transporters (ENTs) compared to the negligible activity mediated by the concentrative nucleoside transporters (CNTs). Particular uptake rates mediated by ENT1 or ENT2 subtypes could be individualized through selective inhibition with 1 μM S-(4-nitrobenzyl)-6-thio-inosine (NBTI) or 2 mM hypoxanthine, respectively [30]. Thus, the uptake mediated by the equilibrative nucleoside transporter-1 (ENT1) was far more significant in comparison to the transport activity mediated by ENT2 in these cells (Fig 1A). Further, we mimicked the effects of diabetes in HK2 cells by exposing them to high D-glucose concentrations. Upon exposure of HK2 to 25mM D-glucose, the ENT1-mediated uptake of adenosine decreased by 50% in comparison to 5mM D-glucose (Fig 1A).

**Fig 2:**
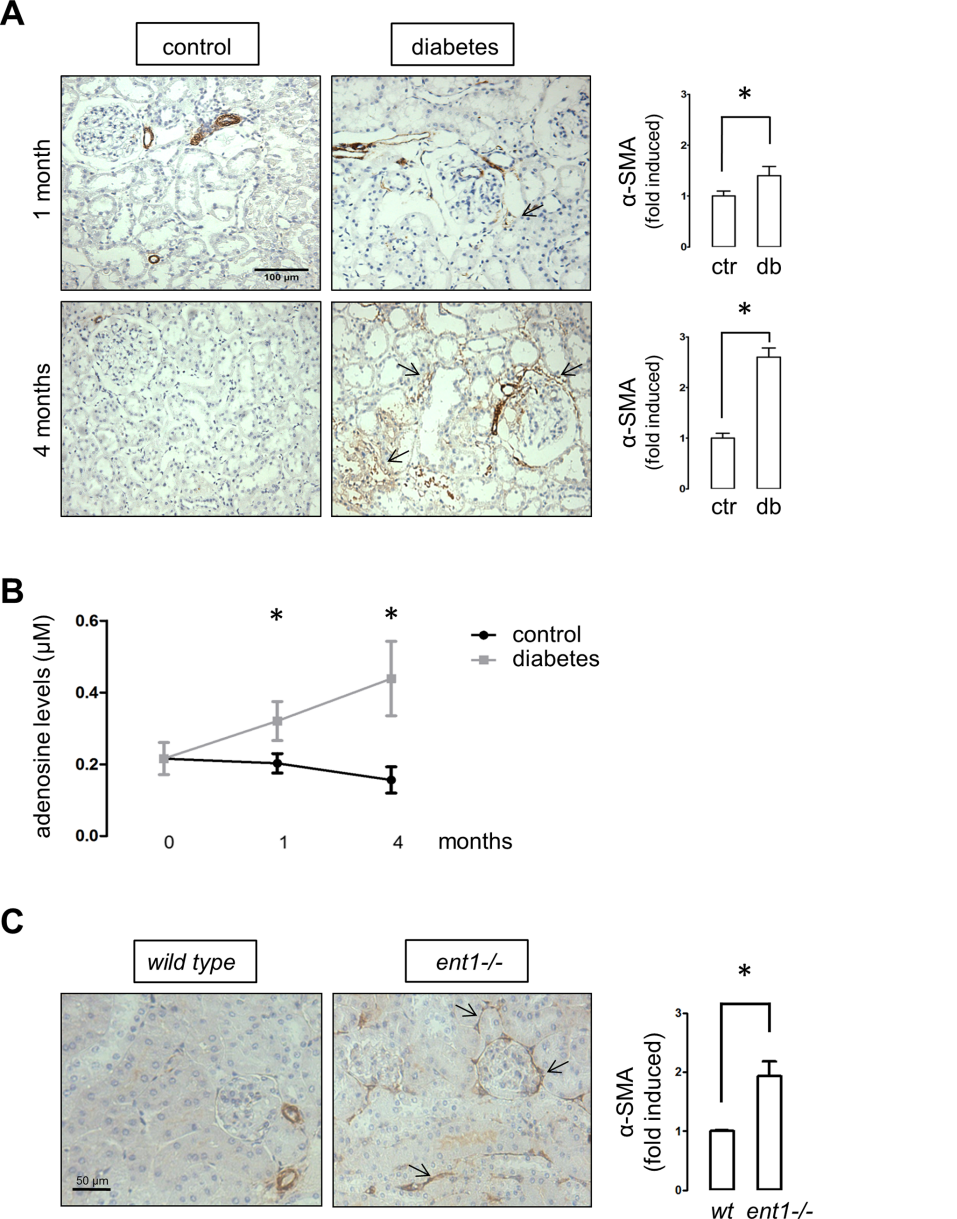
Evidences of renal fibrosis in diabetic rats and *ent1* knockout mice. A. Representative immunohistochemical detection of α-SMA in kidney sections from vehicle- and STZ-treated diabetic rats. The arrows indicate interstitial and periglomerular staining. B. The graph depicts plasmatic adenosine levels in control and diabetic rat groups. * *P* < 0.05 vs control, n = 6. C. Representative immunohistochemical detection of α-SMA in kidney sections from 8 weeks old *ent1-/-* and wild type mice. The graphs (A and C) show quantitative results derived from staining analyses of consecutive field from three animals per group using the UN-SCANIT 2.0 software. Values of control or wild type samples were normalized to 1. * *P* < 0.05 vs control or wild type. Original magnifications 200X.

To further assess the consequences of experimental diabetes on adenosine handling, proximal tubules were isolated from healthy and diabetic rats. Following the induction of experimental diabetes in rats, glycemic levels were raised to values around 450 mg/dl, gaining of mass was depressed and the kidney weight relative to the total body weight was increased which is indicative of kidney hypertrophy (Table 1). The progression of renal injury was evidenced by the detection of increasing levels of proteinuria and urea, manifestly higher than controls animals at 4 months post diabetes induction. In addition, nephropathy manifests with increased uric acid levels, a metabolite generated from purines catabolism (Table 1). Furthermore, an extensive abundance of the alpha-smooth muscle actin (α-SMA) was detected in the cortical area of kidney from diabetic rats following four months of diabetes induction (Fig 2A). Therefore, our analyses in tissue samples from rats following 1 and 4 months post diabetes induction represent early and manifest stages of kidney injury respectively.

**Table 1:** Physiological parameters in streptozotocin-induced diabetic rats.

		1 month		4 months	
		control	diabetes	control	diabetes
***Body weight (gr)***		518 ± 53	315 ± 74 *	593 ± 115	284 ± 120 *
***Glycaemia (mg/dl)***		124 ± 21	449 ± 188 *	133 ± 99	482 ± 80 *
***Relative kidney weight (g/Kg)***	***Left***	6.24 ± 0.6	10.6 ± 1.8 *	5.35 ± 0.3	10.93 ± 1.5 *
	***Right***	5.47 ± 0.7	9.7 ± 1.9 *	5.32 ± 0.8	10.60 ± 1.7 *
***Proteinuria (mg/mg creatinine)***		1.8 ± 0.3	2.3 ± 0.6	2.5 ± 0.6	4.9 ± 0.6 *
***Urinary Urea (mg/mg creatinine)***		47.6 ± 5.9	50.4 ± 8.5	39.1 ± 3.5	87.0 ± 10.2 *
***Urinary Uric Acid (mg/mg creatinine)***		0.079 ± 0.083	0.140 ± 0.09	0.077 ± 0.03	0.172 ± 0.05 *

*, *P* < 0.05 diabetes vs control (n = 6).

Adenosine uptake rates were found to be decreased by diabetes in isolated renal tubules due to a lesser ENT1-mediated activity (Fig 1B), despite the fact that the content of ENT1 in rat renal tissues was not altered by experimental diabetes (Fig 1C). Reduction of adenosine uptake mediated by ENT1 was higher than 70% following one (0.230 ± 0.087 vs 0.060 ± 0.039 pmol • μmoles prot^-1^ • min^-1^, *P* value < 0.01) and four (0.200 ± 0.047 vs 0.053 ± 0.019 pmol • μmoles prot^-1^ • min^-1^, *P* value < 0.01) months from diabetes induction (Fig 1B). Remarkably, the consequence of inhibition of ENT1 activity using NBTI resulted in a marked increase in extracellular adenosine levels in HK2 cells (Fig 1D).

### Adenosine mediates profibrotic changes in epithelial tubule cells

A pivotal correlation was evidenced between the mark of the α-SMA, a sign of profibrotic cells activation, and the increase of plasma levels of adenosine in diabetic rats (Fig 2B). Pearson correlation value r was 0.7934 with *P* value < 0.01 (n = 28). Furthermore, kidneys isolated from *ent1*^-/-^ knockout mice showed higher levels of α-SMA (Fig 2C). Adenosine renal interstitial levels have been found to rise in *ent1*^*-/-*^ mice or animals treated with an inhibitor of its activity [21]. Also, when cells were incubated with dipyridamole to block ENT transport activity, profibrotic changes were induced [23]. These observations suggest a link between altered adenosine handling and profibrotic activation that remains to be elucidated.

**Fig 3:**
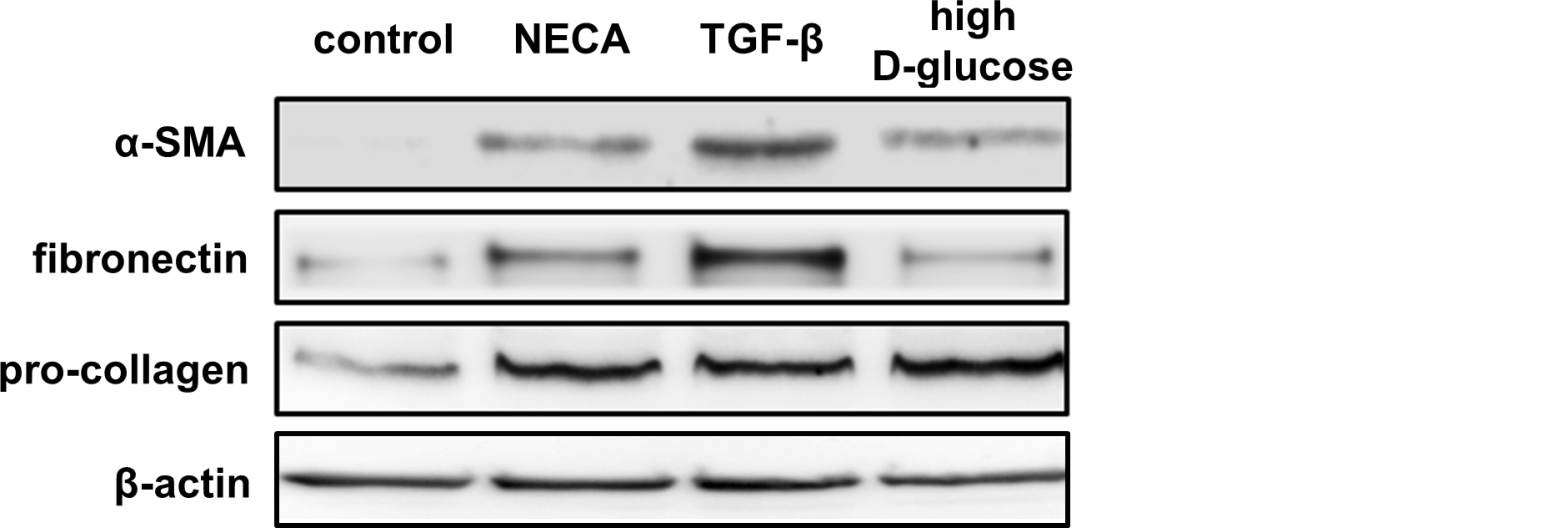
Phenotypic transition of HK2 cells. The induction of profibrotic markers α-SMA, fibronectin and pro-collagen were evaluated by western blot in HK2 cells treated for 24 h with the general agonist of adenosine receptors NECA (5 μM), TGF-β (2 ng/ml) and high D-glucose (25mM). Representative western blots are shown.

The cellular effects of adenosine are mediated by the P_1_ family of purinergic receptors. The four subtypes of adenosine receptors were found at RNA and protein levels in HK2 cells. The exposure of HK2 cells to the general adenosine receptors agonist NECA induced the expression of the profibrotic markers fibronectin, α-SMA and procollagen type I (Fig 3). These effects were similar to those observed using high D-glucose (25mM) or TGF-β, recognized inducers of profibrotic phenotypic transition in epithelial cells (Fig 3).

**Fig 4:**
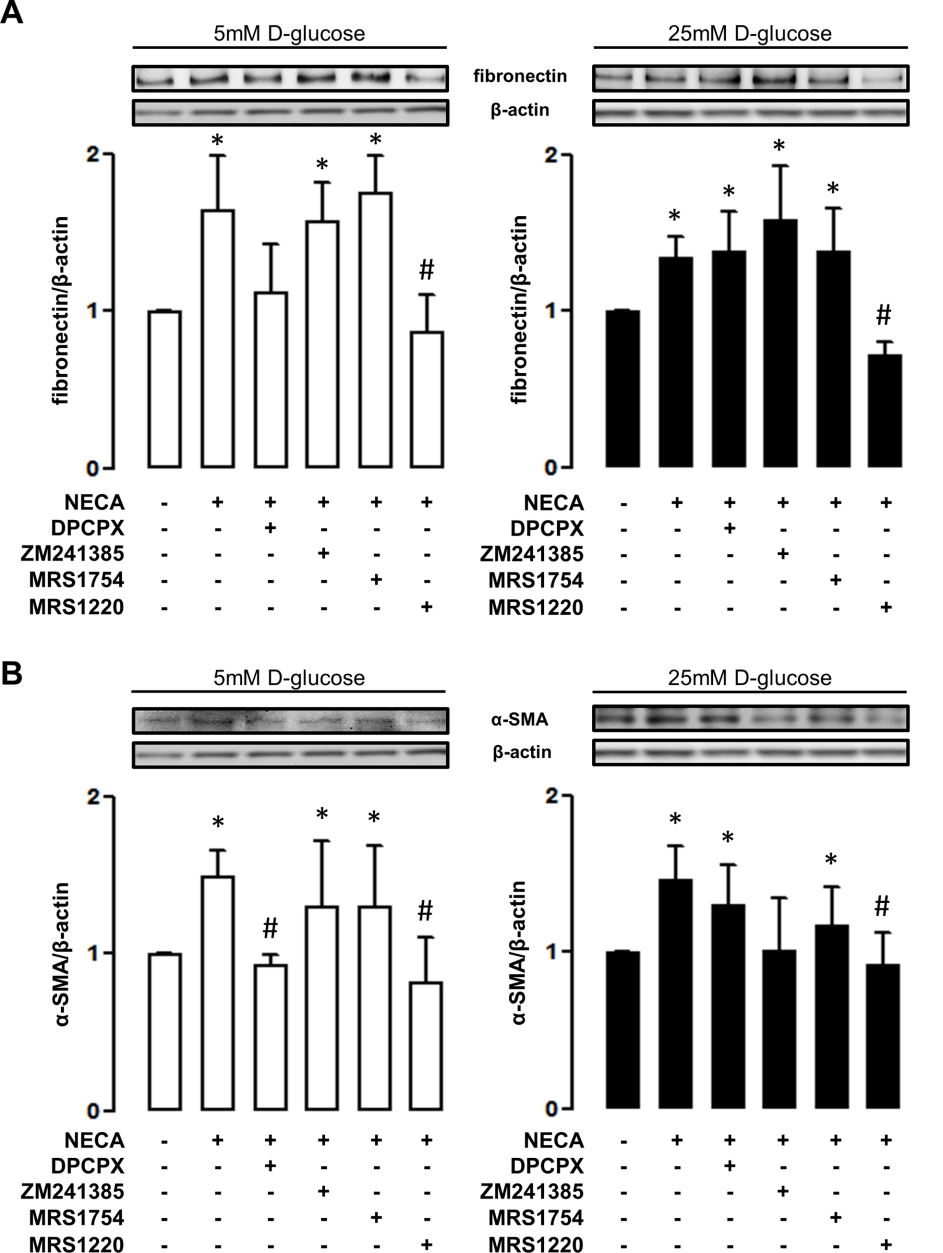
An antagonist of adenosine A_3_ receptor blocks NECA-induced profibrotic markers. The particular adenosine receptor subtype involved in profibrotic activation was recognized by using selective pharmacological antagonists for each receptor subtype, in conjunction with the general agonist NECA (5 μM). The induction of the markers fibronectin (A) and α-SMA (B) in HK2 cells, was evaluated by western blots. Selective antagonists were DPCPX (30nM) for A_1_, ZM241385 (10nM) for A_2A_, MRS1754 (50nM) for A_2B_ and MRS1220 for A_3_ (10nM) receptor subtypes. The upper images show representative western blot detections of marker content in total protein extracts (50 μg) from treated HK2 cells. The blocking effect was assayed in HK2 cell cultures in 5mM (left white bars graphs) or 25mM (right black bars graphs) D-glucose. The graphs represent the mean ± SD of the ratio between immune signals of fibronectin or α-SMA vs β-actin. The ratio in HK2 cells without any pharmacological modulator was normalized to 1. * *P* < 0.05 versus untreated cells, # *P* < 0.05 versus NECA, n = 6.

**Fig 5:**
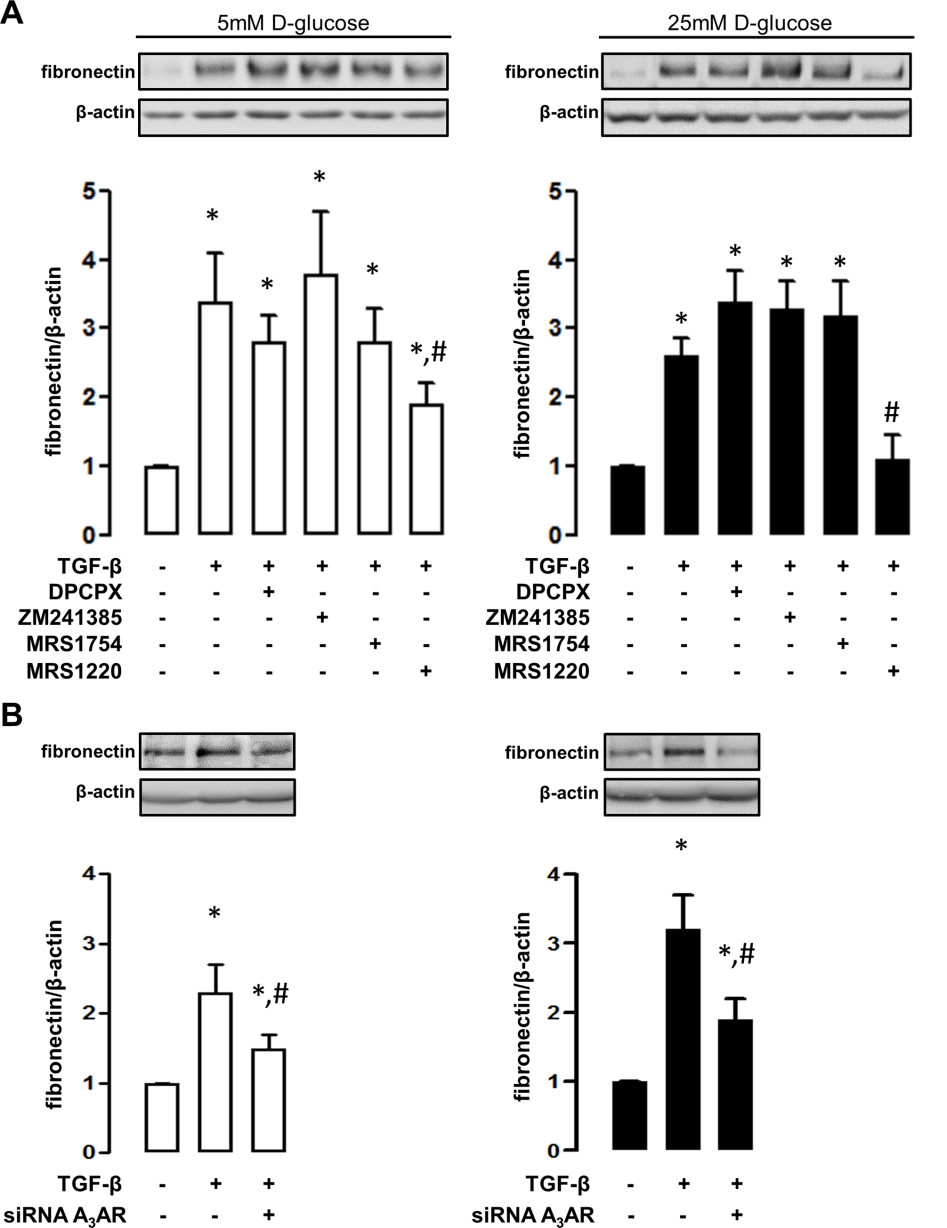
An antagonist of adenosine A_3_ receptor blocks profibrotic activation induced by TGF-β. A. The induction of the marker fibronectin was evaluated by western blots in HK2 cells upon exposure to TGF-β. The contribution of a particular adenosine receptor subtype in TGF-β-induced cell activation, was recognized by using selective pharmacological antagonists. Selective antagonists were DPCPX (30nM) for A_1_, ZM241385 (10nM) for A_2A_, MRS1754 (50nM) for A_2B_ and MRS1220 for A_3_ (10nM) receptor subtypes. B. The particular contribution of the adenosine A_3_ receptor was evidenced by knocking down the expression of the receptor using siRNA (siRNA A_3_AR). The upper images show representative western blot detections of fibronectin content in total protein extracts (50 μg) from treated HK2 cells. The blocking effect was assayed in HK2 cells cultures 5mM (left white bars graphs) or 25mM (right black bars graphs) D-glucose. The graphs represent the mean ± SD of the ratio between immune signals of fibronectin vs β-actin. The ratio in HK2 cells without TGF-β treatment was normalized to 1. * *P* < 0.05 versus untreated cells, # *P* < 0.05 versus TGF-β, n = 5.

To characterize the adenosine receptor subtypes involved in profibrotic activation, HK2 cells were exposed to a set of selective pharmacological antagonists in conjunction with the general agonist NECA. As shown in Fig 4, the induction of fibronectin and α-SMA mediated by NECA can be blocked using MRS1220, the antagonist of adenosine A_3_ receptor subtype, independent of the concentrations of D-glucose in the medium (Fig 4). In addition, the antagonist of the A_1_ receptor subtype blocked the induction of markers in cells exposed to 5mM D-glucose but it was unable to reproduce these effects in cells exposed to a high D-glucose environment (Fig 4). Interestingly, the profibrotic activation using TGF-β can be also blocked by MRS1220 in this cell line in both 5 and 25 mM D-glucose conditions (Fig 5A). Further demonstration of the role of the adenosine A_3_ receptor subtype was obtained from siRNA experiments. In addition to prevent the induction of fibronectin mediated by NECA and high D-glucose (S1 Fig), the knockdown of the A_3_ receptor subtype prevented HK2 cell transition in 5 and 25 mM D-glucose conditions TGF-β induced (Fig 5B).

### In vivo evaluation of an adenosine A_3_ receptor antagonist in diabetic rats

**Fig 6:**
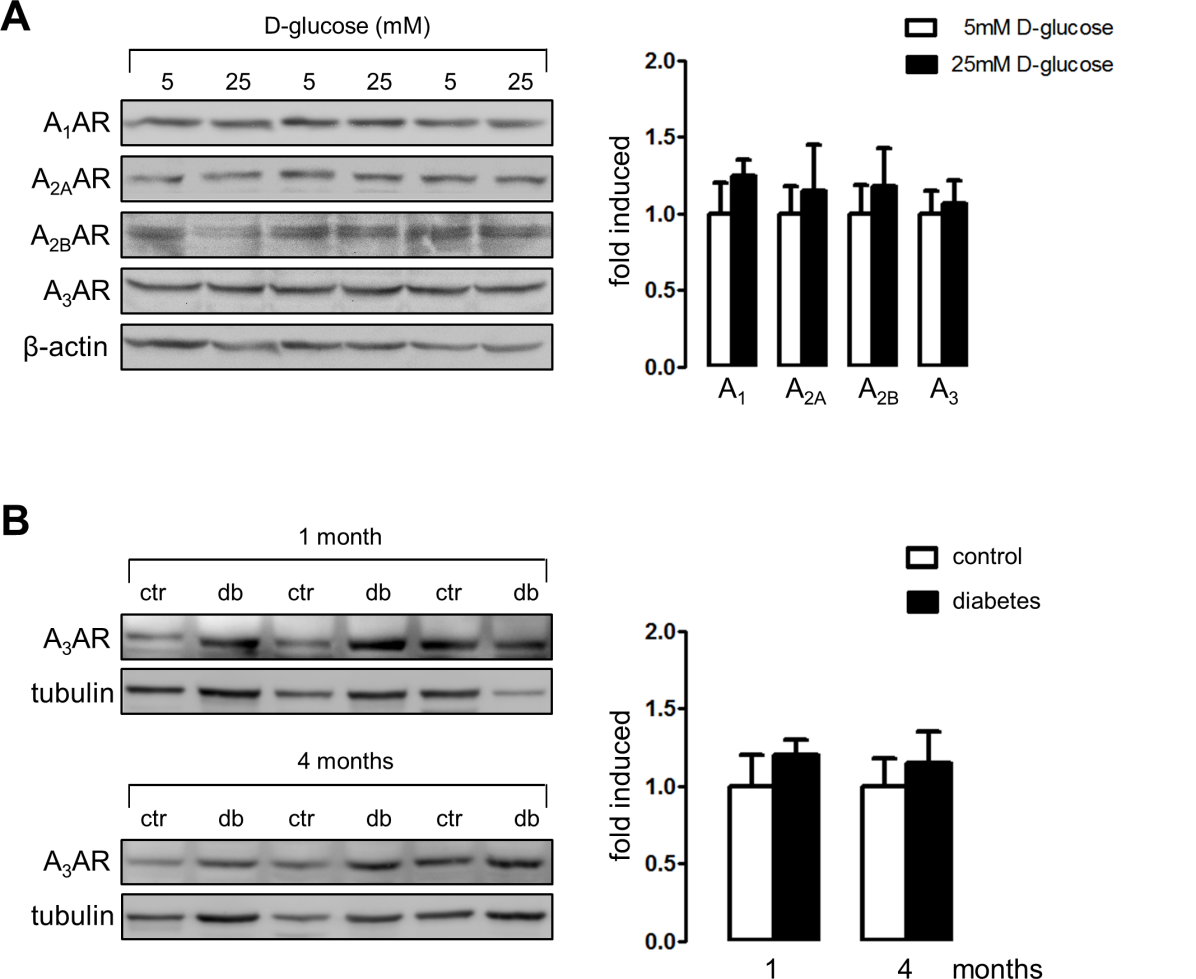
Effect of high D-glucose and experimental diabetes on the adenosine receptors content. A. The effect of D-glucose exposure (24 h) on the protein levels of adenosine receptor subtypes A_1_, A_2A_, A_2B_ and A_3_ in HK2 cells was evaluated by western blot. Representative images of western blots of total protein extracts from cells exposed to 5 and 25 mM D-glucose are shown. B. The effect of STZ-induced diabetes on adenosine A_3_ receptor content was evaluated by western blot of total protein extracts from tubules isolated from control (ctr) and diabetic (db) rats following 1 or 4 months post-induction. The graphs show quantitative analysis of immune signals. The means in controls were normalized to 1. A: n = 6, B: n = 3.

**Fig 7:**
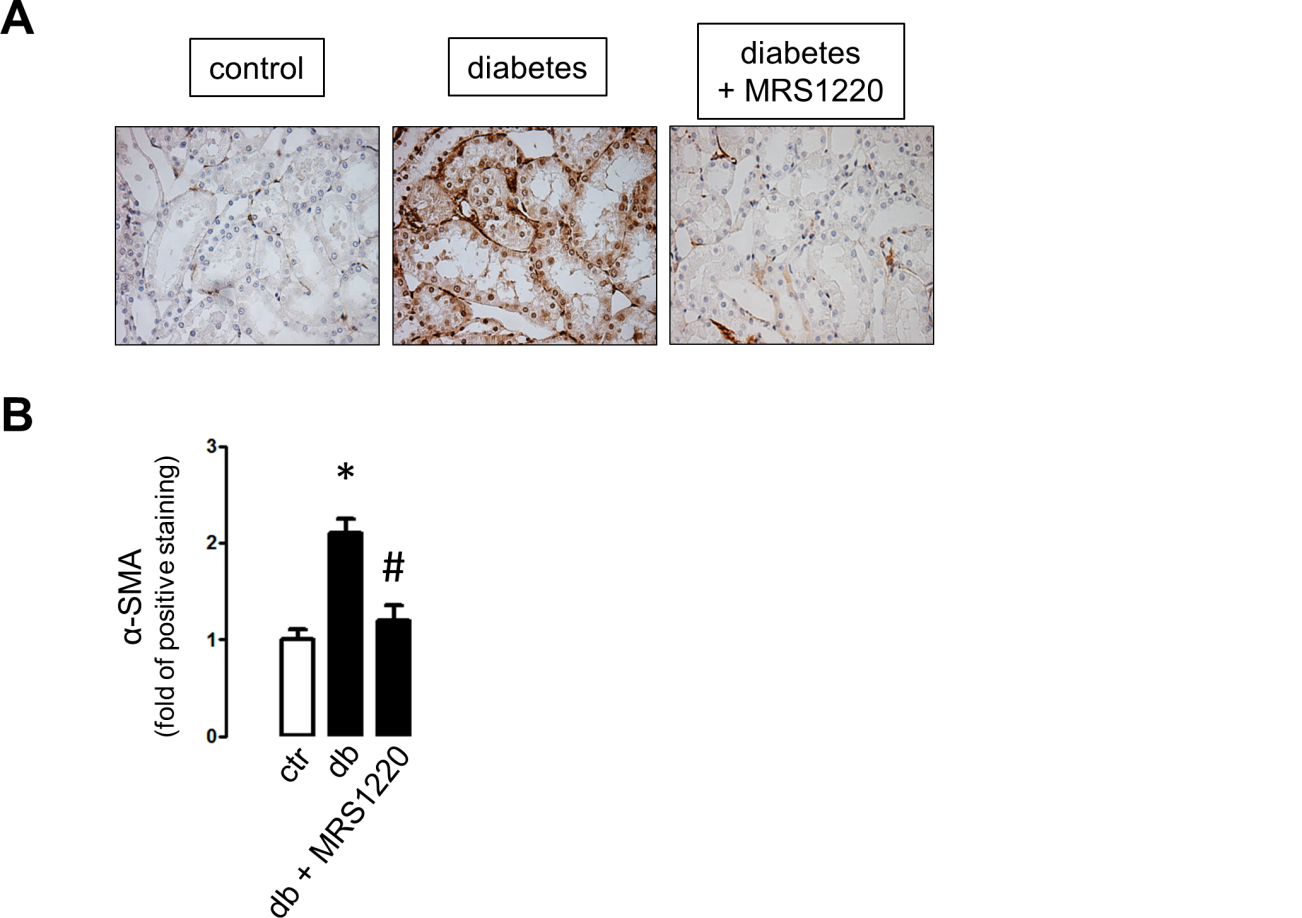
In vivo effects of pharmacological inhibition of A_3_AR. Diabetic rats were treated with the adenosine A_3_ receptor antagonist MRS1220 from days 31 to 60 post diabetes induction, A. Representative inmunohistochemical detection of α-SMA in cortical areas of kidney sections from untreated (control), diabetic (vehicle-treated rats) and MRS1220-treated diabetic rats. Original magnifications 400X. B. The graph shows quantitative results derived from staining analyses of consecutive field from three animals per group using the UN-SCANIT 2.0 software. Values of control samples were normalized to 1. * *P* < 0.05 vs control.

The examination of the content of the adenosine receptors in HK2 cells by western blot analysis indicates that the total levels were not affected by the exposure of cells to different D-glucose concentrations (Fig 6A). Furthermore, the content of adenosine receptors in purified tubule extracts did not change following 1 or 4 months post diabetes induction in rats (Fig 6B). Therefore, the possibility to be pharmacologically modulated is conceivable. Following one month post diabetes induction, diabetic rats were treated with MRS1220 or vehicle for 4 weeks. Immune staining of α-SMA was significantly reduced in renal cortical tubules from MRS1220-treated animals compared to vehicle-treated diabetic rats (Fig 7). Treatment with the A_3_ receptor antagonist did not affect the increased plasma adenosine levels in diabetic rats at this stage (data not shown).

### Evaluation of ENT1 and adenosine A_3_ receptor in human diabetic nephropathy

**Fig 8:**
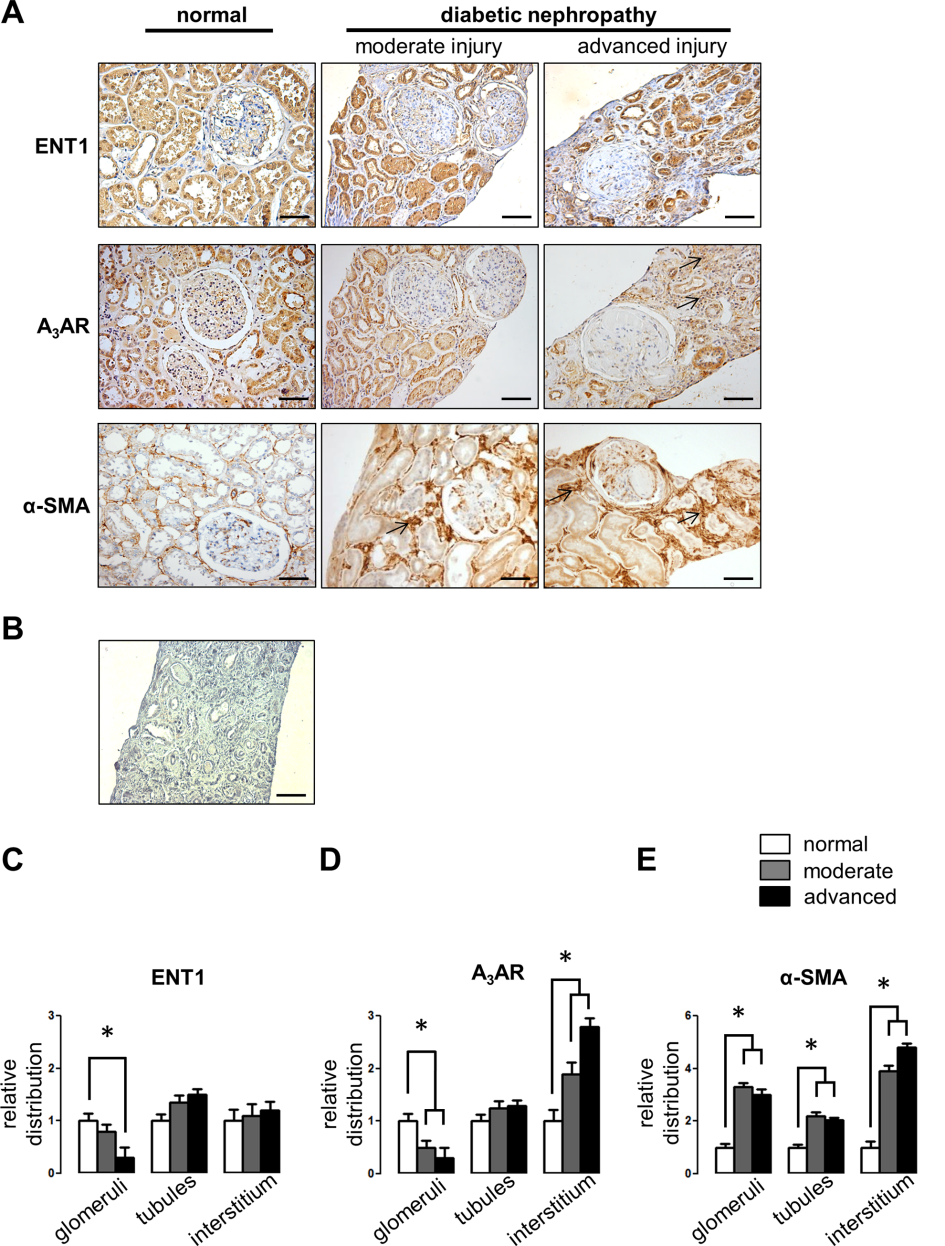
Expression of ENT_1_ and A_3_AR in human kidney. A. Immunohistochemical detection of ENT1 and A_3_ receptor proteins were carried out in human kidney sections from non-diabetic normal tissue and biopsies from diabetic nephropathy patients. Selected images denote representative progressive stages of renal injury probed by the content of α-smooth muscle actin (α-SMA) and pathological analysis. Arrows indicate interstitial distribution of the immune signal. Original magnification 200x. Scale bars 50μm. B. Representative negative control without primary antibody. Quantitative image analyses of immune staining for ENT1 (C) A_3_AR (D) and α-SMA (E) in human biopsies. The graphs show quantitative results derived from staining analyses in defined areas (at least 5 glomeruli, 10 tubules and 10 interstitium per sample) using the UN-SCANIT 2.0 software. Human biopsies from 9 diabetic nephropathy patient and 3 controls are individualized in S1 Table. Values in normal tissue samples were normalized to 1. * *P* < 0.05 vs normal.

Whereas, the STZ-treated diabetic rat model permits the early evaluation of moderate kidney injury, human DN progresses with an extensive renal fibrosis. To further evaluate the expression of ENT1 and the adenosine A_3_ receptor throughout the progression of human diabetic kidney disease, immune detection of both proteins were carried out in biopsies from normal, moderate and advanced kidney injury. Clinical parameters in patients are shown in S1 Table. The distribution of α-SMA was used to show progression of kidney fibrosis in biopsies (Fig 8). In the most severe renal tubule-interstitial fibrosis damage, glomerulosclerosis with low cellularity and loss of tubular structures was evident. The ENT1 expression was mainly restricted to tubular epithelium and some glomerular cells. This pattern was maintained with the progression of renal injury. The adenosine A_3_ receptor was located in the epithelial tubule cells, glomerular cells and Bowman’s capsule in normal tissue. Along with the lower glomerular cellularity observed in advanced stages of renal injury, both the content of ENT1 and A_3_AR were decreased in renal glomeruli. In contrast, the tubulointerstitial distribution of the adenosine A_3_ receptor subtype was shown increased in human biopsies throughout the progression of kidney injury (Fig 8).

## Discussion

This study is the first show a link between profibrotic activation of renal epithelial tubule cells with the activity of the adenosine A_3_ receptor in the context of diabetic renal disease. Thus, the purpose of our research was i) to demonstrate the role of high glucose on altered nucleoside handling in proximal tubule epithelial cells, ii) correlate lower nucleoside uptake activity mediated by ENT1 with increased adenosine levels and iii) to connect increased ligand levels with cellular profibrotic effects mediated by low affinity adenosine receptors. We contribute to the emerging notion that implicates a chronic increase of adenosine with fibrotic processes. Indeed, experimental models have associated such elevated levels of nucleoside and adenosine receptor activity with the progress of hepatic, dermal and pulmonary fibrosis, and recently with chronic kidney disease [31–33].

ENT1 is the main nucleosides transporter in proximal tubules [34] and responsible for the basolateral uptake of adenosine [21]. Extensive distribution of ENT1 throughout the kidney indicates that this transporter plays a role in adenosine signaling beyond nucleoside and nucleobase homeostasis [20,35]. While, decreased ENT1 activity, capable of increasing adenosine levels, has been described as renoprotector in an acute kidney injury model using tissue ischemia and reperfusion [21], the chronic reduction of nucleoside uptake could underlay DN pathogenic fibrosis. Certainly, long term hyperglycemia concurs with decreased ENT1 activity and increasing levels of adenosine in our experimental model. In turn, autocrine/paracrine adenosine signaling mediates fibrotic phenotypic changes in epithelial tubule cells. This injurious loop was impeded by knocking down the A_3_ receptor in HK2 cells and by using a pharmacological antagonist in diabetic rats. These findings suggest that adenosine receptor blockers may be useful as new therapeutic approach.

There are several evidences showing that glucose levels play a role in the control of ENTs activity [30,36–38]. Some reports recognize insulins action of counteracting decreased nucleoside uptake activity imposed by elevated D-glucose, through ENT1 activity rescue and increasing ENT2-mediated transport [30,36–38]. Probably, these facts could link the deficient insulin signaling and hyperglycemia in renal cells with increasing levels of the nucleoside and the pathogenesis of DN. It is worth noting that in glomeruli isolates from diabetic rats, extracellular adenosine levels were considerably increased in comparison to controls however differences were not seen for other adenine nucleotides [22]. Only sodium-independent uptake activity mediated by ENT1 was inhibited. Probably, it became the main engine for increasing extracellular adenosine, despite the fact that slight changes on AMP hydrolyzing activity have been previously described [22].

The particular increase of plasma adenosine levels in DN patients was described in recent years [9,10], linking the progression of renal injury with adenosine signaling. Using diverse experimental models it has been further suggested that adenosine signaling and receptor induction are common pathogenic pathways in chronic kidney injury [33]. Thus, the adenosine deaminase knockout (*ada-/-*) mice model, exhibiting augmented levels of the nucleoside because of impeded metabolism, shows renal dysfunction and sclerosis. In addition, Dai and collaborators demonstrated that development of renal fibrosis generated in *ada*^*-/-*^ animals can be avoided using an A_2B_AR antagonist [33]. Promising outcomes were also obtained on renal fibrosis in mice caused by angiotensin II or unilateral ureteral obstruction (UUO) [33]. Furthermore, the diabetic glomerular alterations in STZ-treated rats were ameliorated using an antagonist of A_2B_AR [22], regardless of the recent proposal that A_2B_AR has a vascular protective effect in a model of DN [39]. Interestingly, it has recently been described the implication of the adenosine A_3_ receptor subtype in mediating fibrosis in the UUO experimental model [28], so the antagonist LJ-1888 was able to ameliorate tubule-interstitial fibrosis. Consequently, such protective effects of antagonists of low affinity receptors A_2B_ and A_3_ could come from the inhibition of the pathogenic effects of adenosine differentially occurring in renal cells types since, the induction of the A_2B_ receptor subtype has been related to podocyte cell dysfunction, increased TGF-β release and diabetic glomerulosclerosis [20,22] while here, we showed that the A_3_ receptor may be associated with profibrotic activation of tubular epithelial cells.

The location of the A_3_AR subtype in the kidney has not been conclusively determined. We found that the localization of A_3_AR was widely distributed in epithelial tubule cells. In addition, we detected the A_3_AR protein in glomerular cells. Pawelczyk et al [40] described that membrane-associated A_3_AR protein levels increased by 70% in diabetic kidney cortex and decreased by 80% in medulla. Such increase may not be ascribed to proximal tubule cells because we did not observe changes in the receptor content due to diabetes. Notably, the receptor content was extended to tubulointerstitium in biopsies from DN patients with advanced progression of injury. This fact suggests that with the progression of the disease the interstitium was enriched with A_3_AR positive cells that could direct interstitial fibrosis. One of the mechanisms involved in renal fibrosis is the infiltration of macrophages which secret several cytokines underlying fibroblast activation and myofibroblast generation [41,42]. In this context, the anti-inflammatory effect of A_3_AR agonists has been previously suggested [43,44]. On the other hand, epithelial proximal tubule cells seem to be capable of transdifferentiate to a primitive mesenchymal phenotype (epithelial to mesenchymal transition, EMT) in response to certain physiological cues, conducted predominantly by TGF-β [45,46]. In essence, has been proposed these cells appear to be capable of altering their morphology, expressing myofibroblastic markers, and migrating through basement membrane into the interstitium, thus joining the pool of activated fibroblasts. It is suggested that EMT contributes up to one-third of the interstitial cells population that produce fibrotic mediators and extracellular matrix [47]. This process has been recognized in biopsies of patients with DN [48] and strong correlations were established between the EMT occurrence, interstitium expansion and collagen deposition [49,50]. In fact, EMT, myofibroblastic transdifferentiation, proteinuria and the decline in renal function are strongly correlated thus, supporting α-SMA immunostaining to monitor renal fibrosis in diabetic patients [49,50]. Thus, the exact source of A_3_AR positive cells and their role to renal fibrosis remains to be a debate.

Notably, the induction of α-SMA and fibronectin mediated by TGF-β in HK2 cells were blocked by the A_3_AR antagonist, suggesting a crosstalk between adenosine and the cytokine intracellular signaling. Consequently, it was suggested that the anti-fibrotic effect of the A_3_AR antagonist LJ-1888 in the presence of TGF-β up-regulation in the UUO rats kidneys, may account for MAP kinases inactivation [28]. It has been described that the adenosine A_1_ and A_3_ receptor subtypes share some signaling properties such as both receptors are coupled to G_i_ protein and could trigger MAP kinases activation [24,51]. This property could be evident when antagonizing A_1_ and A_3_ receptor subtypes, both preventing HK2 cell transdifferentiation under low D-glucose levels. However, the particular signaling property that implicates A_3_AR with TGF-β mediated profibrotic activation, is a novel topic that remain to be elucidated.

Collectively, our data link the mechanism mediating the increased adenosine levels with the activity of adenosine A_3_ receptor. This study supports the evaluation of preclinical studies to assess the efficacy of this new alternative for the treatment of DN targeting renal fibrosis.

## Supporting Information

S1 Fig Adenosine A_3_ receptor knockdown blocks fibronectin induction mediated by NECA and high D-glucose. A. The content of the adenosine A_3_ receptor was evaluated by western blot in HK2 cells transfected with commercially available and validated short interfering RNAs (siRNA) from Ambion (catalogue number AM16708). Typically transfection using 100 pmol of selective siRNA decreased A_3_AR by 80%. B. The induction of the EMT marker fibronectin was evaluated by western blot in HK2 cells upon exposure to NECA (5μM) and 5mM (control) or 25mM (HG) D-glucose. The particular contribution of the A_3_AR was evidenced by knocking down the expression of the receptor using siRNA (siRNA A_3_). The graph represents the mean ± SD of the ratio between immune signals of fibronectin vs tubulin. The ratio in HK2 cells in 5mM D-glucose treatment was normalized to 1. # *P* < 0.05 versus NECA or HG, n = 6.
(TIF)

S1 Table Clinical parameters of patients. (TIF)
