# Supplementary figures and images for: Reduced Adenosine Uptake and Its Contribution to Signaling that Mediates Profibrotic Activation in Renal Tubular Epithelial Cells: Implication in Diabetic Nephropathy
Source: PLoS One. 2016 Jan 25;11(1):e0147430. doi: 10.1371/journal.pone.0147430 (PMC4726618; doi:10.1371/journal.pone.0147430)

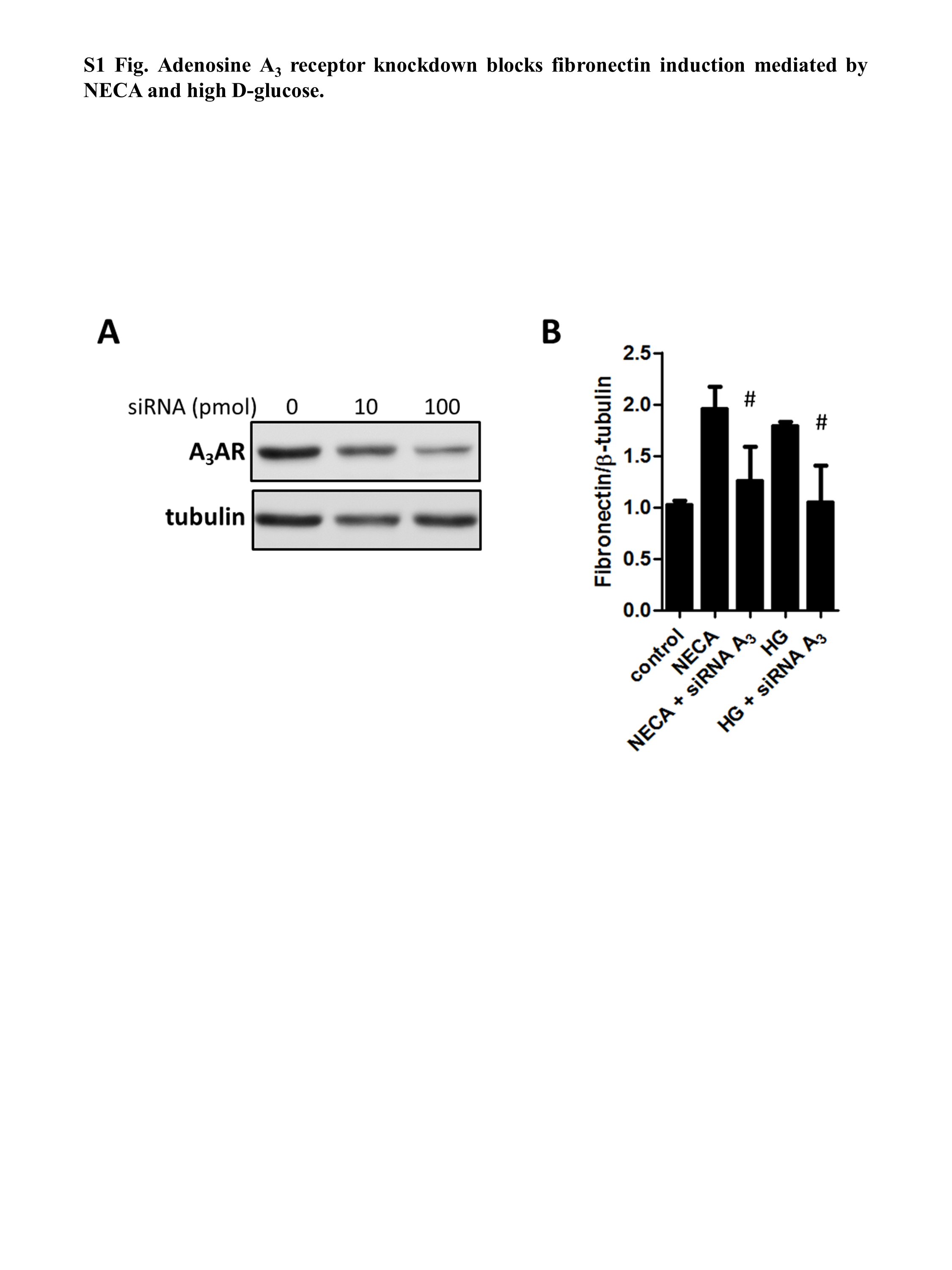

Supplement: S1 Fig — A. The content of the adenosine A3 receptor was evaluated by western blot in HK2 cells transfected with commercially available and validated short interfering RNAs (siRNA) from Ambion (catalogue number AM16708). Typically transfection using 100 pmol of selective siRNA decreased A3AR by 80%. B. The induction of the EMT marker fibronectin was evaluated by western blot in HK2 cells upon exposure to NECA (5μM) and 5mM (control) or 25mM (HG) D-glucose. The particular contribution of the A3AR was evidenced by knocking down the expression of the receptor using siRNA (siRNA A3). The graph represents the mean ± SD of the ratio between immune signals of fibronectin vs tubulin. The ratio in HK2 cells in 5mM D-glucose treatment was normalized to 1. # P < 0.05 versus NECA or HG, n = 6. (TIF) [file pone.0147430.s001.tif]

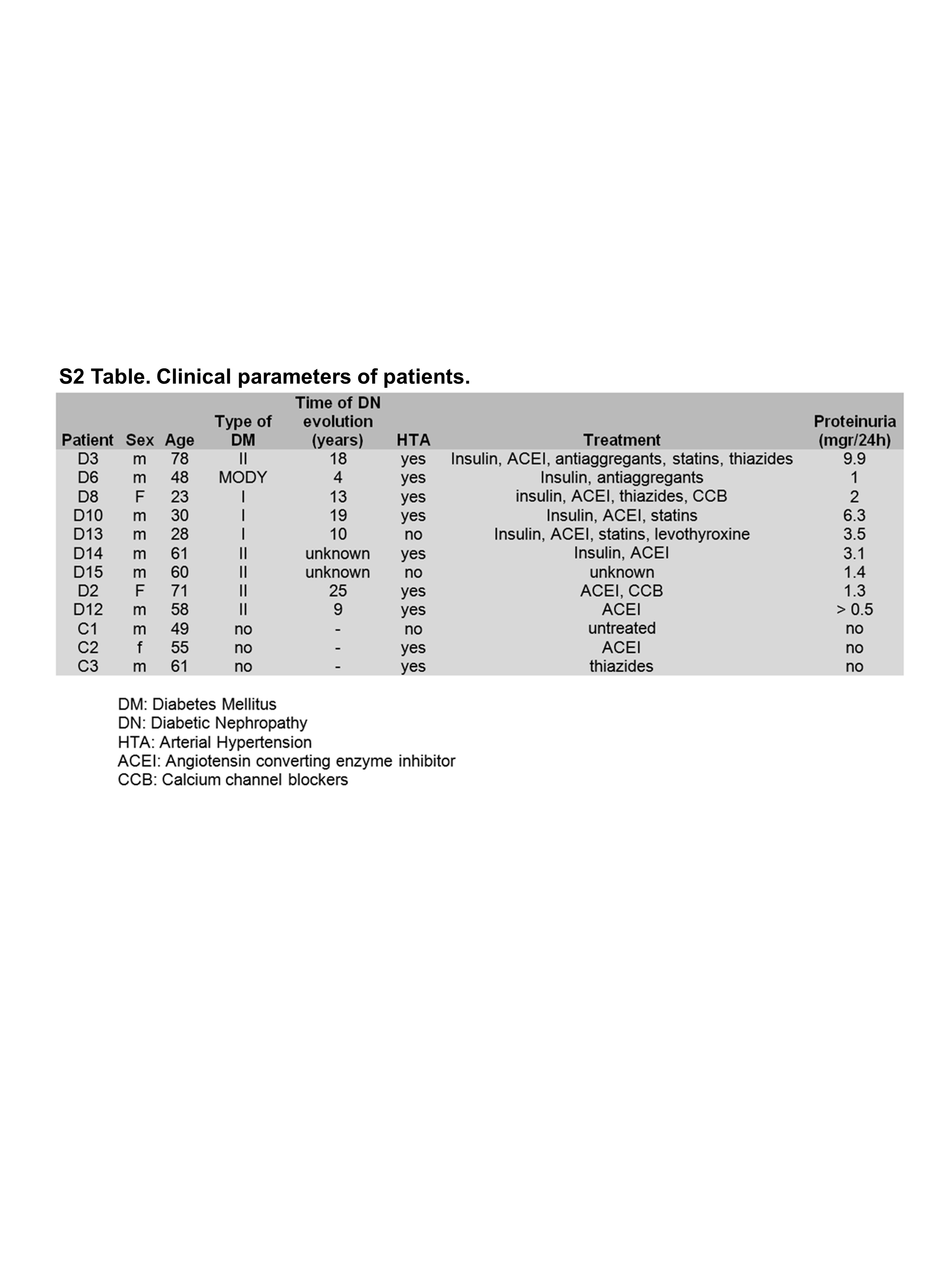

Supplement: S1 Table — (TIF) [file pone.0147430.s002.tif]
